# Estrogen receptors promote NSCLC progression by modulating the membrane receptor signaling network: a systems biology perspective

**DOI:** 10.1186/s12967-019-2056-3

**Published:** 2019-09-11

**Authors:** Xiujuan Gao, Yue Cai, Zhuo Wang, Wenjuan He, Sisi Cao, Rong Xu, Hui Chen

**Affiliations:** 1 Department of Pharmacology, School of Basic Medicine, Tongji Medical College, Huazhong University of Science and Technology, No. 13, HangKong Road, Wuhan, 430030 Hubei China; 2 The Key Laboratory for Drug Target Researches and Pharmacodynamic Evaluation of Hubei Province, Wuhan, 430030 Hubei China

**Keywords:** Estrogen receptors, Non-small cell lung cancer, Membrane receptor signaling network, Cancer systems biology

## Abstract

**Background:**

Estrogen receptors (ERs) are thought to play an important role in non-small cell lung cancer (NSCLC). However, the effect of ERs in NSCLC is still controversial and needs further investigation. A new consideration is that ERs may affect NSCLC progression through complicated molecular signaling networks rather than individual targets. Therefore, this study aims to explore the effect of ERs in NSCLC from the perspective of cancer systems biology.

**Methods:**

The gene expression profile of NSCLC samples in TCGA dataset was analyzed by bioinformatics method. Variations of cell behaviors and protein expression were detected in vitro. The kinetic process of molecular signaling network was illustrated by a systemic computational model. At last, immunohistochemical (IHC) and survival analysis was applied to evaluate the clinical relevance and prognostic effect of key receptors in NSCLC.

**Results:**

Bioinformatics analysis revealed that ERs might affect many cancer-related molecular events and pathways in NSCLC, particularly membrane receptor activation and signal transduction, which might ultimately lead to changes in cell behaviors. Experimental results confirmed that ERs could regulate cell behaviors including cell proliferation, apoptosis, invasion and migration; ERs also regulated the expression or activation of key members in membrane receptor signaling pathways such as epidermal growth factor receptor (EGFR), Notch1 and Glycogen synthase kinase-3β/β-Catenin (GSK3β/β-Catenin) pathways. Modeling results illustrated that the promotive effect of ERs in NSCLC was implemented by modulating the signaling network composed of EGFR, Notch1 and GSK3β/β-Catenin pathways; ERs maintained and enhanced the output of oncogenic signals by adding redundant and positive-feedback paths into the network. IHC results echoed that high expression of ERs, EGFR and Notch1 had a synergistic effect on poor prognosis of advanced NSCLC.

**Conclusions:**

This study indicated that ERs were likely to promote NSCLC progression by modulating the integrated membrane receptor signaling network composed of EGFR, Notch1 and GSK3β/β-Catenin pathways and then affecting tumor cell behaviors. It also complemented the molecular mechanisms underlying the progression of NSCLC and provided new opportunities for optimizing therapeutic scheme of NSCLC.

## Background

Lung cancer is the most common cancer and is the leading cause of cancer deaths for both men and women worldwide [1, 2]. Epidemiological data suggest that sex hormones are associated with the incidence, therapeutic response and clinical outcomes of lung cancer [3]. And in most cases, sex hormones play a role in lung cancer by binding to their corresponding receptors. ERs are important sex hormone receptors, which are proposed to affect the development and progression of lung cancer, particularly in NSCLC.

It is generally agreed that ERs are expressed both in the cytoplasm and nucleus of NSCLC cells and exert their effects through both the genomic and non-genomic mechanisms [4, 5]. The genomic mechanism is mediated by estrogen-responsive elements or AP-1 [6, 7]. Non-genomic mechanism involves crosstalks between ERs and growth factor receptor pathways, such as epidermal growth factor receptor (EGFR) pathway [8]. However, the role of ERs in NSCLC prognosis still remains controversial. Some studies have reported that high expression of estrogen receptor α (ERα) and/or estrogen receptor β (ERβ) correlates with poor prognosis in NSCLC [9–11]. There are also some reports suggesting that ERs are favorable prognostic factors for NSCLC patients [12, 13]. These controversies indicate that the molecular mechanisms of ERs in NSCLC are probably more complicated than reported. Therefore we will attempt to reconsider the effect of ERs in NSCLC from a more comprehensive perspective.

Recently, there has been accumulating evidence supporting that cancer is a complex systemic disease involving dysregulation of multiple pathways and loss of homeostasis at multiple levels [14–16]. Given the complexity of cancer, it can be inferred that the effects of ERs on NSCLC are likely to be mediated by multiple pathways interacted with each other rather than some individual targets. Therefore, it may be reasonable to explore the role of ERs in NSCLC from a perspective of cancer systems biology [17, 18], which is an emerging approach to investigate the complexity of cancer origin and evolution from a holistic view. Bioinformatics method and high-throughput database facilitate a more comprehensive understanding of the influence of ERs variation on genomes and pathways in NSCLC. And computational model can be used to illustrate the interaction among the signaling networks, which are composed of genes and pathways influenced by ERs. Furthermore, prediction capabilities of systemic models will help solve practical problems such as acquired resistance and therapeutic strategy optimization.

In this study, we intend to integrate bioinformatics method, experimental approach, computational modeling and IHC analysis, to explore the role of ERs in NSCLC from the perspective of cancer systems biology.

## Materials and methods

### Cell lines and cell culture

The human NSCLC cell lines PC9, H1299, A549, H1975, HCC827 were purchased from the Cell Bank of Type Culture Collection of the Chinese Academy of Sciences (Shanghai, China). The Gefitinib-resistant cell line PC9/G was generated as described previously [19]. All cell lines were cultured in recommend medium supplemented with 10% FBS and 1% penicillin–streptomycin and were cultured at 37 °C in a 5% CO_2_ incubator as protocols described. All cell lines were authenticated by STR DNA profiling.

### Cell viability assay

Cells were seeded into 96-well plates at a density of 3000 per well. After 24 h incubation, cells were treated with different concentration of β-Estradiol (17β-E2, Sigma-Aldrich, USA) for 72 h. 10% CCK-8 (Zoman Bio, China) diluted in normal culture medium was added to each well and incubated for an additional 1.5 h. The absorbance was measured spectrophotometrically at 450 nm. Each experiment was performed at least three times independently.

### Cell transfection

ERα siRNA (sc-29305, sc-44204) and ERβ siRNA (sc-35325) were purchased from Santa Cruz Biotechnology (Dallas, USA). Cells were incubated in 6-well plates supplemented with antibiotic-free normal growth medium until the cells were 60–80% confluent. Negative control siRNA (si-NC), si-ERα or si-ERβ (100 nM) were mixed with Lipofectamine^®^ 3000 Reagent (Invitrogen, USA), and were added to the siRNA transfection medium (Opti-MEM, Gibco, USA). This transfection mixture was added to each plate for 6 h and then replaced by normal growth medium according to the manufacturer’s instructions.

### Cell apoptosis analysis

Cells were seeded into 6-well plates and treated with Gefitinib (AstraZeneca) after transfection or combined with Fulvestrant (ICI 182,780, Sigma-Aldrich) for 48 h. Cells in each well were harvested, washed and resuspended in 1× binding buffer, and then stained with Annexin V-FITC and PI (Sungene Biotech, China) for 10 min in the dark, respectively. Data acquisition was performed on a flow cytometry (Becton–Dickinson, USA) with the CellQuest software (BD Biosciences, USA).

### Cell migration and invasion assay

After transfection, cells were resuspended in serum-free medium containing Gefitinib or not and were seeded into the upper chambers of Transwell inserts (Corning Costar, USA) with (for invasion assays) or without (for migration assays) Matrigel (BD Biosciences, USA). Normal culture medium was added into each of the bottom chambers. After 24 h (for migration assays) or 48 h (for invasion assays) incubation, cells on the surface of the bottom chamber were stained with 0.1% crystal violet (Google biotechnology, China). The stained cells were photographed and counted under an inverted microscope at 400× magnification.

### Western blot analysis

Cells were harvested after transfection or drug treatment. Total intracellular protein was extracted, quantified and denatured. Equal amounts of protein were fractionated by SDS-PAGE gels and transferred onto PVDF membranes. The membranes were first incubated with the corresponding primary antibodies overnight and then incubated with horseradish peroxidase-conjugated secondary antibodies for another 1 h. Protein bands were visualized and analyzed using chemiluminescence system (Pierce Biotechnology, USA) and Gel-Pro Analyser (Media Cybernetics Inc., USA). Details about antibodies were listed in Additional file 1: Table S1.

### Human tissue samples and IHC analysis

NSCLC tissue microarrays (Cat. No. HLugA180Su02; National Human Genetic Resources Sharing Service Platform, Shanghai, China) annotated with clinical information were collected from 93 patients who underwent surgical resections from July 2004 to June 2009. This study was approved by the Institutional Review Board for Clinical Research of Tongji Medical College, Huazhong University of Science and Technology, with informed consent from all patients. Protein expressions of EGFR, ERα, ERβ and Notch1 were detected by IHC analysis proceeded as the manufacturer’s instructions. The median values of final IHC scores were applied as the cut-off criterion. Antibodies used in this analysis were list in Additional file 1: Table S2.

### Dataset and bioinformatics analysis

The gene expression data of NSCLC tumor tissues were download from the UCSC Xena (dataset ID: TCGA.LUNG.sampleMap/HiSeqV2; samples: 1129; unit: log2(norm_count + 1)) [20, 21]. In this study, we would focus on the expression characteristics of ESR1 and ESR2 genes in NSCLC tumor tissues, so data from 110 normal tissue samples were excluded. Genes with an average expression value < 2 were excluded because the very low-expression genes might not participate in a specific process in the cell. The final data set was an expression matrix of 17,489 genes from 1019 tumor samples. The R package “limma” [22] was used for differential expression analysis. Fold change > 2 and *p* < 0.05 was utilized to identify differently expressed genes (DEGs). Gene Ontology (GO) [23] and Kyoto Encyclopedia of Genes and Genomes (KEGG) pathway [24] enrichment analysis were performed using the DAVID online tool [25], *p* < 0.05 was set as the cut-off criterion. Visualization of the DEGs in this dataset was carried out by using the ‘clustergram’ function in MATLAB^®^ software (version: R2016a, 64-bit), the dissimilarity metric was Euclidean distance.

### Computational modeling the molecular signaling network

The laws that governed the biochemical reactions used in our model were based on Henri-Michaelis–Menten kinetics, mass action law and irreversible constant flux law. The biological dynamic signaling transduction process inside the NSCLC cells was modeled by a set of ordinary differential equations. The MATLAB toolbox PottersWheel [26] was used for model constructions, parameter estimations and simulations.

### Statistical analysis

Statistical analysis was performed using GraphPad Prism 7 (GraphPad Software, USA). χ^2^ test and two-tailed Student’s t-test were applied to determined statistical significance. Survival analysis was estimated by the Kaplan–Meier method.

## Results

### GO and KEGG analysis revealed that ERs affected some membrane receptor signaling pathways

**Fig. 1:**
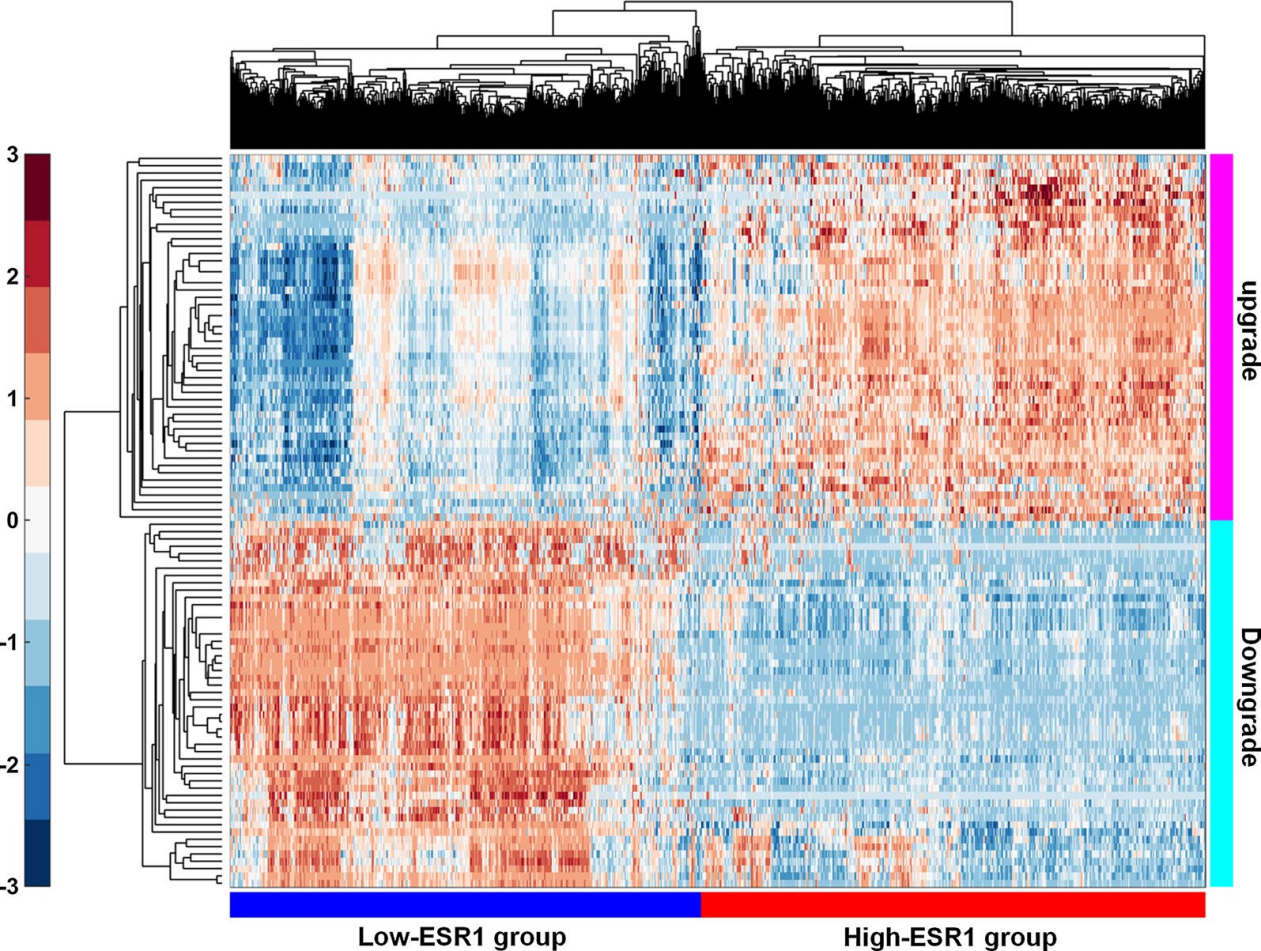
The top 100 DGEs (The top 50 up-regulated genes and 50 down-regulated genes) between the low-ESR1 group (N = 510) and high-ESR1 group (N = 509) were analyzed by hierarchical clustering. Each row represented a single gene and each column represented a tissue sample. Red indicated relatively high expression and green indicated relatively low expression

To outline the effect of ERs in NSCLC, the gene expression data of NSCLC in TCGA database were analyzed. Using the mRNA level of ESR1, which encoded the ERα protein, as the phenotypic label, 1019 tumor tissue samples were divided into the high-ESR1 group (N = 509) and the low-ESR1 group (N = 510), the median value of ESR1 expression level was used as cut-off criterion. Similarly, for ESR2, which encoded the ERβ protein, these 1019 samples were also divided into the high-ESR2 group (N = 509) and the low-ESR2 group (N = 510). Differential expression analysis showed that there were 1237 DEGs between the low- and high-ESR1 group, of which 769 genes were upgraded in the high-ESR1 group and 468 genes were downgraded. For ESR2, there were 102 DEGs between the low- and high-ESR2 group, of which 95 genes were upgraded in the high-ESR2 group and 7 genes were downgraded. Hierarchical clustering showed systematic variations in the expression of DEGs in NSCLC (Fig. 1).

The DEGs were uploaded to DAVID to identify overrepresented GO categories and KEGG pathways. Results of enrichment analysis for 769 upgraded and 468 downgraded DEGs in the high-ESR1 group were listed in Table 1 and Additional file 2. The upgraded DEGs in the high-ESR1 group were mainly enriched in the terms of signal transduction, immune and inflammatory responses, cell adhesion, receptor binding and activation. The downgraded DEGs in the high-ESR1 group were mainly enriched in the terms of transcriptional regulation, oxidation–reduction process, calcium ion binding, CYP450-related metabolism. For ESR2, no significant term was found for enrichment analysis of these 7 downgraded DEGs. The upgraded DEGs in the high-ESR2 group were mainly enriched in the terms of cell adhesion, embryonic limb morphogenesis, epithelial cell differentiation, calcium ion binding, structural molecule activity and GABAergic synapses (Table 2 and Additional file 2). For cell component of GO analysis, both of DEGs in ESR1 and ESR2 group were mainly enriched in plasma membrane and extracellular space. Taken together,the results suggested that variations of ESR1/2 expression might affect many important molecular events and pathways, especially cell communication, including receptor activation, signal transduction, cell adhesion, immune response, which might predominate in ESR1/2-mediated regulation in NSCLC.

**Table 1:** GO and KEGG pathway enrichment analysis of DEGs in the high-ESR1 group

	Category	Term	Count	p value	Genes (partial)
Upgrade	BP	Signal transduction	69	1.3E−04	DLC1, GNA14, CLDN3, WISP2, CDKL2, TNFRSF10C, CCR6, C3, KIT, GPRC5A, CCL20, CD4, ITK
	BP	Immune response	60	7.8E−19	CHIA, SUSD2, HLA-DMA, CXCR5, SPN, NCR3, TNFRSF10C, CCR2, C3, CXCL2, CCL20, CD4
	BP	Cell adhesion	49	1.1E−10	CXCR3, WISP2, ICAM1, NCAM2, ITGAL, VCAM1, COMP, CD2, THBS4, DPT, TNXB, CASS4
	CC	Integral component of membrane	262	2.5E−10	CCR5, ROR1, MST1R, CYP1B1, ICM1, MMP13, CD22, ABCA3, MUC1, VCAM1
	CC	Plasma membrane	255	5.7E−21	CADM3, AQP1, BTK, ADAM8, ROS1, PIK3CG, CDHR4, CCR5, RAB17, KDR, ICAM1, C3, CD4, MRC1
	CC	Extracellular exosome	182	9.3E−16	PDGFD, MUC1, BMP3, WISP2, ICAM1, AGT, FGG, C3, CD4, CPM
	MF	Calcium ion binding	41	4.1E−03	MMP28, FAT4, COMP, ADAM8, CAPN9, CDHR4, PCDHAC1
	MF	Protein homodimerization activity	37	3.6E−02	CADM3, PTGS2, KIT, CD2, CEACAM5, MUC13, FLT3, S100B, CCR2, AOC3
	MF	Receptor binding	30	3.4E−05	CADM3, C3, BLK, BTK, PGR, FGA, RSRO3, TNFSF8, CCL13
	KEGG	Cell adhesion molecules (CAMs)	33	9.8E−15	ITGAL, CADM3, CLDN9, ITGB2, HLA-DMA, ITGAM, VCAM1, CD2, SELPLG, SPN, ICAM1, PTPRC
	KEGG	Cytokine-cytokine receptor interaction	29	3.1E−06	GDF5, CXCL2, IL21R, CXCR5, LTB, CSF1R, TNFRSF17, TNFSF8, CCR7, CCL13, CCR6, CD40LG
	KEGG	Hematopoietic cell lineage	28	2.1E−16	HLA-DRB1, FCER2, KIT, ITGB3, ITGAM, MS4A1, CD4, CSF3R, CR1, FLT3, CD1A
Downgrade	BP	Positive regulation of transcription from RNA polymerase II promoter	37	1.9E−03	FGFR2, WNT3A, E2F7, SOX2, TP63, JAG1, BARX1, NRG1, DMRT1, CHP2, SIX2, GAL, HMGA2, ITGA6, BMP7
	BP	Negative regulation of transcription from RNA polymerase II promoter	26	1.7E−02	FGFR2, E2F7, SOX2, MAGEA1, TP63, NRARP, TRIM29, DMRT1, VAX1, HMGA2, NR0B1, DLX1, FOXE1, TBX18
	BP	Oxidation–reduction process	25	3.1E−03	CYP26A1, OSGIN1, ADH7, ALDH3A1, SESN3, FMO6P, CYP4F3, NOS2, AKR1C1
	CC	Extracellular exosome	84	2.6E−03	WNT3A, RASSF9, SERPINB5, PI3, CNTN1, RAB3B, UGT1A6, KRT5, TGM1, LGALS7, DSC1, IGFBP2, PSAT1
	CC	Extracellular region	55	1.2E−03	WNT3A, JAG1, NRCAM, NRG1, CLCA2, TMPRSS11A, FGFR2, ADH7, FBN2, WNT2B, SOST, BMP7, IGFBP2
	CC	Extracellular space	52	9.9E−05	WNT3A, FGF12, NRG1, MMP10, SERPINB5, FGFBP1, LGALS7, KRT31, WNT2B, SOST, IGFL1, BMP7, IGFBP2
	MF	Structural molecule activity	32	7.7E−15	JAG1, KRT5, CLDN20, SPRR1A, KRT16, SPRR3, CSTA, ADD2
	MF	Calcium ion binding	31	8.6E−04	NELL1, JAG1, NECAB2, CDH8, ANXA8, RPTN, FAT2, TGM3, FBN2, S100A2, CDHR1, CABYR, MMP10, DSC1
	MF	Transcription factor activity, sequence-specific DNA binding	31	4.4E−02	E2F7, SOX2, TP63, ZIC1, BARX1, HOXC8, FOXD1, PITX1, TRIM29, SIX2, DLX2, FOXE1, TBX18, TCF15
	KEGG	Metabolism of xenobiotics by cytochrome P450	15	2.8E−10	GSTA1, CYP2S1, ADH7, UGT1A1, ALDH3A1, GSTM3, UGT1A8, UGT1A3, UGT2A1, AKR1C1
	KEGG	Neuroactive ligand-receptor interaction	14	5.5E−03	GABRR1, PTH2R, CHRM3, P2RY1, S1PR5, LPAR3, GPR50, CHRNB2, ADRA2B, HTR2C, GABRQ
	KEGG	Drug metabolism—cytochrome P450	12	1.4E−07	GSTA1, UGT1A7, UGT1A10, GSTM3, UGT1A9, UGT2A1, ADH7, UGT1A1, ALDH3A1

The top 3 terms containing the largest number of DEGs from Biological Processes (BP), Cell Components (CC), Molecular Functions (MF) and KEGG Pathways are listed in the table, respectively. The last column shows partial genes enriched in each term, the complete list of genes and terms can be found in Additional file 2

**Table 2:** GO and KEGG pathway enrichment analysis of DEGs in the high-ESR2 group

Category	Term	Count	p value	Genes
BP	Cell adhesion	6	3.6E−02	CLCA2, COL7A1, PKP1, ADAM23, DSC3, COL4A6
BP	Embryonic limb morphogenesis	5	1.9E−05	HOXC10, DLX6, DLX5, BMP7, HOXD10
BP	Epithelial cell differentiation	5	1.7E−04	RHCG, DLX6, DLX5, UPK1B, BMP7
CC	Extracellular exosome	20	1.5E−02	COCH, GDA, KRT6B, LGALS7, KRT13, CALB1, A2ML1, LGALS7B, CD19, KRT74, NEB, PKP1, KRT5, RHCG, CALML3, DSG3, UPK1B, MS4A1, SPRR3, SERPINB13
CC	Integral component of plasma membrane	12	2.7E−02	EPHA7, CLCA2, CD19, SLCO1A2, RHCG, ADAM23, PTPRZ1, GABRA3, CDHR1, NTRK2, UPK1B, MS4A1
CC	Keratin filament	4	7.8E−03	KRT74, KRT6B, KRT5, KRT13
MF	Calcium ion binding	8	3.1E−02	CALML3, DSG3, CDHR1, NELL2, DSC3, CALB1, PCDH19, CACNA1B
MF	Sequence-specific DNA binding	7	2.2E−02	HOXC10, DLX6, SOX2, FOXE1, DMRT2, HOXD10, HOXD11
MF	Structural molecule activity	6	3.8E−03	KRT74, KRT5, SPRR2A, UPK1B, SPRR3, KRT13
KEGG	GABAergic synapse	3	3.8E−02	GABRA3, HAP1, CACNA1B

The top 3 terms containing the largest number of DEGs from Biological Processes (BP), Cell Components (CC), Molecular Functions (MF) and KEGG Pathways are listed in the table, respectively. A complete list of genes and terms can be found in Additional file 2

Moreover, DEGs enriched in the above terms of GO and KEGG pathway were involved in many membrane receptor signaling pathways. As shown in Tables 1, 2 and Additional file 2, DEGs were involved in the following pathways: growth factor signaling pathway (such as FGF12, FGFR2, IGFBP2, PDGFD and PIK3CG), Wnt/GSK/β-Catenin pathway (such as FZD10, LRP4, MARK1, SFRP4, WISP2, WNT2B and WNT3A) and Notch pathway (such as Jag1, MSI1, NRARP and TP63). These results indicated that ESR1/2 might directly or indirectly regulate these pathways, which were also important oncogenic signals in NSCLC.

### ERs induced cell proliferation, migration, invasion and apoptosis escape

As shown in the results of bioinformatics analysis, ESR1/2 might regulate many genes and pathways involved in the development and progression of NSCLC. Therefore, before investigating the molecular mechanisms of ERs in NSCLC, we first verified the promote role of ERs in NSCLC at cellular level by detecting the effects of ERs on NSCLC cell phenotypes.

To assess the effects of ERs on cell proliferation, the viability of cells treated with 17β-E2 was detected by CCK assay. The proliferation of PC9/G, PC9 and H1299 cells was increased after 17β-E2 stimulation, while the proliferation of A549, H1975 and HCC827 cells was not affected by 17β-E2 (Additional file 3: Fig. S1A). ERs expression in NSCLC cells was also detected. ERα and ERβ were highly expressed in PC9/G, PC9 and H1299 cells, while ERs expression were relatively low in A549, H1975 and HCC827 cells (Additional file 3: Fig. S1B). The results suggested that high expression of ERs induced NSCLC cells proliferation after 17β-E2 stimulation. It also indicated that the proliferative effect of 17β-E2 was ERs-dependent. Hence we chose PC9/G and H1299 cell lines, which with high expression of ERs, for further study.

**Fig. 2:**
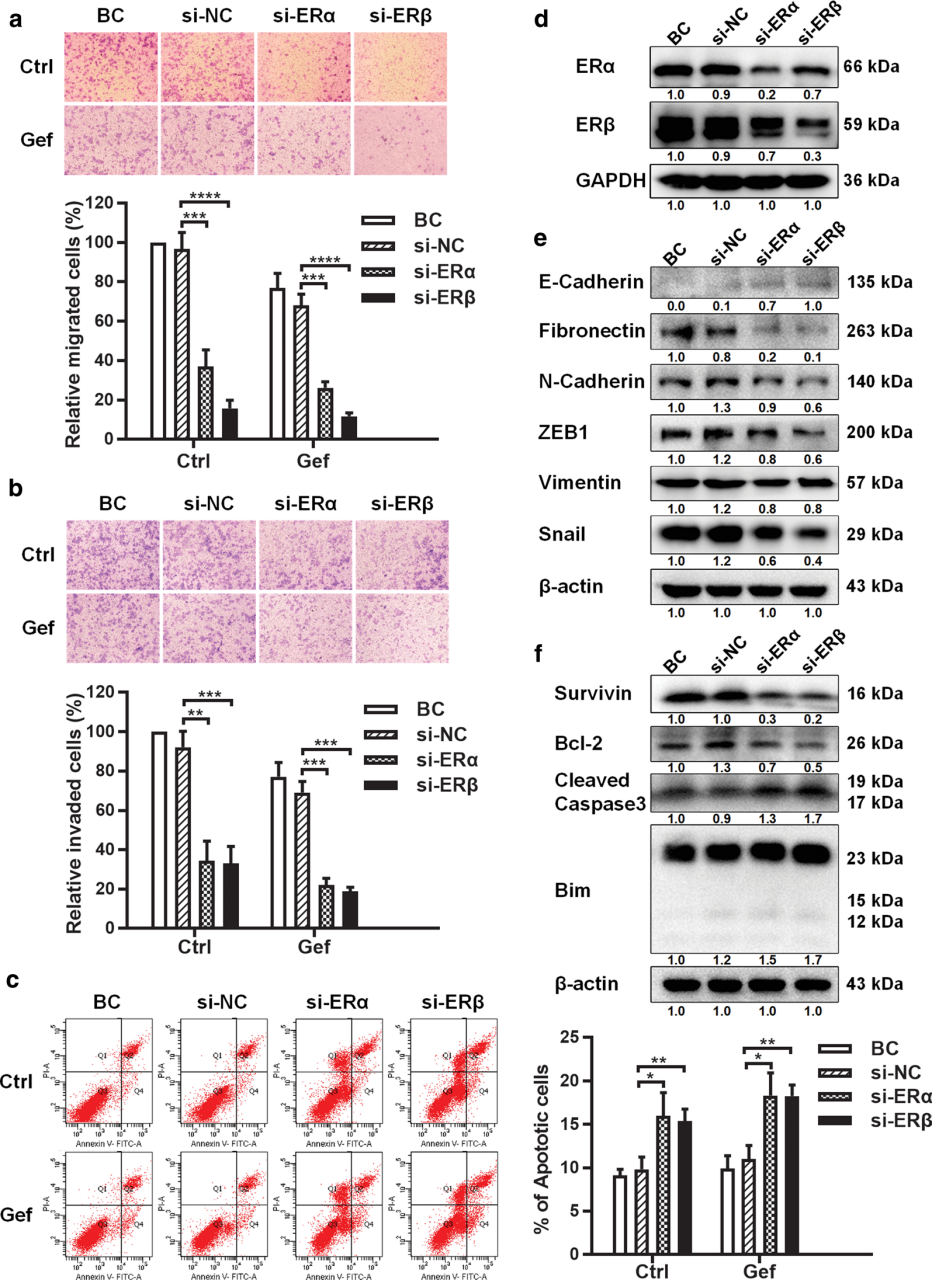
Effects of ERs silencing on NSCLC cell migration, invasion and apoptosis. **a–c** PC9/G cells were transfected with si-NC or si-ERs for 24 h and then treated without or with Gefitinib (20 μM) for 48 h. **a** Cell migration and **b** invasion capacity were measured by Transwell assays. **c** Cell apoptosis amount was determined by flow cytometry analysis. **d**–**f** PC9/G cells were transfected with si-NC or si-ERs for 48 h. The relative expression levels of ERα and ERβ (**d**), migration and invasion associated proteins (**e**), apoptosis associated proteins (**f**) were analyzed by western blot. All experiments were repeated at least three times. *p*-values vs. si-NC were estimated using two-tailed unpaired Student’s t-test, *p < 0.05, **p < 0.01, ***p < 0.001, ****p < 0.0001

PC9/G and H1299 cells were transfected with si-ERα or si-ERβ. After silencing ERs, ERα and ERβ expression was inhibited (Fig. 2d and Additional file 4: Fig. S2D), PC9/G and H1299 cell migration and invasion (Fig. 2a, b and Additional file 4: Fig. S2A, B) were inhibited, and cell apoptosis was increased (Fig. 2c and Additional file 4: Fig. S2C). The effects of ERs silencing on key molecular markers associated with cell migration, invasion and apoptosis were also detected. The results showed that the expression of mesenchymal markers N-Cadherin, Fibronectin, ZEB1, Vimentin and Snail was decreased by ERs silencing, and the expression of epithelial markers E-Cadherin was restored (Fig. 2e and Additional file 4: Fig. S2E). ERs silencing also decreased the expression of the anti-apoptotic proteins Survivin and Bcl-2, and increased the expression of the pro-apoptotic proteins Cleaved Caspase3 and Bim (Fig. 2f and Additional file 4: Fig.S2F). The results suggested that the reduction of cell migration and invasion caused by ERs silencing was probably due to the suppression of epithelial–mesenchymal transition (EMT) process. It also suggested that ERs silencing could induce NSCLC cell apoptosis by regulating the expression of anti- and pro-apoptotic proteins.

### ERs regulated Notch1 and GSK3β/β-Catenin pathway

**Fig. 3:**
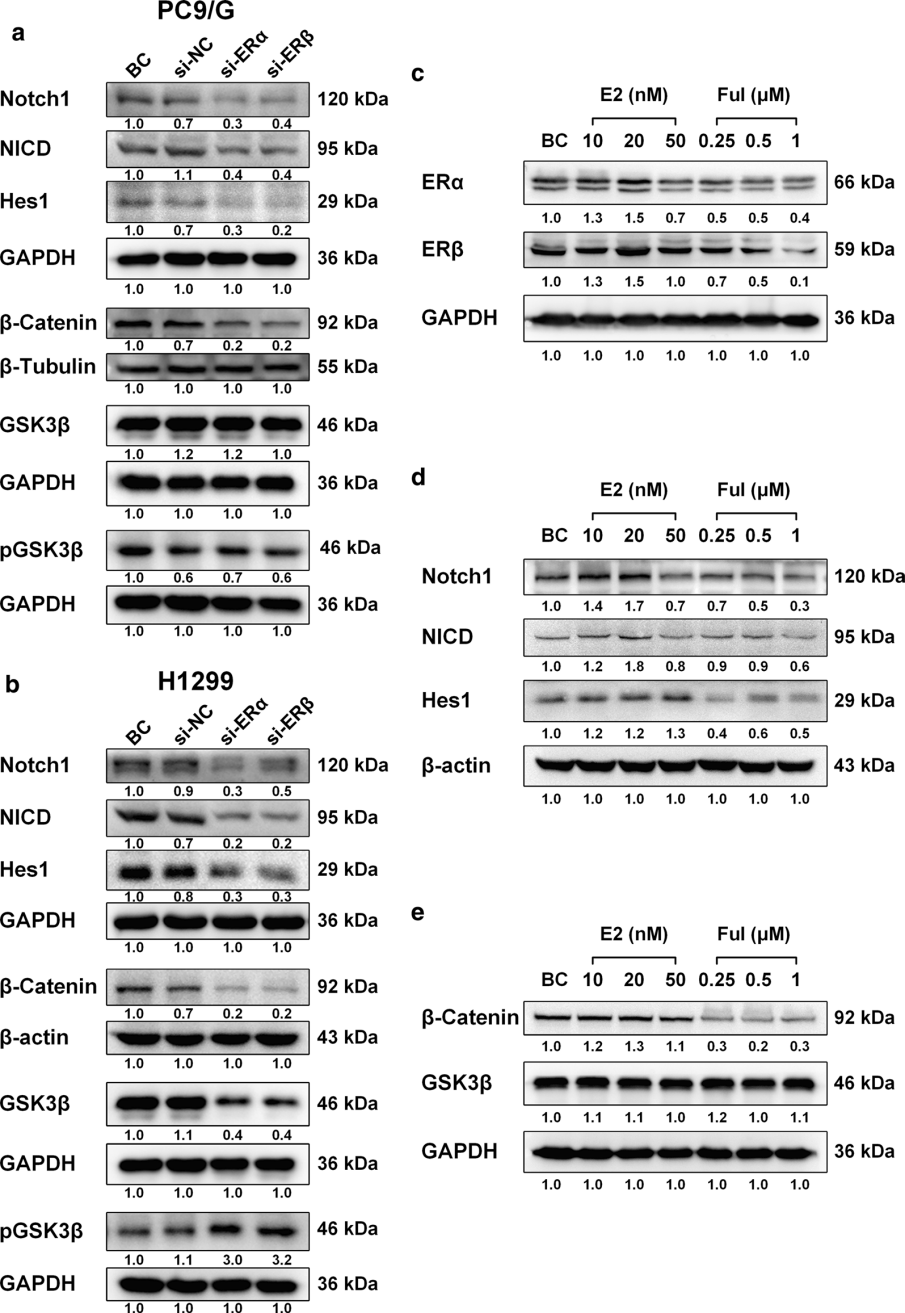
Effects of ERs on key members of Notch1 and GSK3β/β-Catenin pathways. **a**, **b** PC9/G and H1299 cells were transfected with si-NC or si-ERs for 48 h. The relative expression levels of Notch1, NICD, Hes1, β-Catenin, GSK3β and pGSK3β in PC9/G (**a**) and H1299 cells (**b**) were analyzed by western blot. **c**–**e** PC9/G cells were treated with different concentrations of 17β-E2 (E2) and Fulvestrant (Ful) for 12 h. The relative expression levels of ERα and ERβ (**c**), Notch1, NICD and Hes1 (**d**), β-Catenin and GSK3β (**e**) were analyzed by western blot. All experiments were repeated at least three times

The effects of ERs on some representative carcinogenesis-related membrane receptor pathways were detected next. EGFR is likely to interact with ERs has been reported [8]. Besides EGFR, we further investigated whether ERs regulated Notch1 pathway and GSK3β/β-Catenin pathway, which was downstream of the EGFR and Wnt pathways. After ERs silencing, the expression of Notch1 (transmembrane domain of Notch1 receptor), NICD (intracellular domain of Notch1 receptor), Hes1 and β-Catenin was downregulated in both PC9/G (Fig. 3a) and H1299 cells (Fig. 3b). The results suggested that ERs activated Notch1 pathway and inhibited β-Catenin degradation. GSK3β expression was decreased and GSK3β phosphorylation was elevated by ERs silencing in H1299 cells (Fig. 3b). However, the expression and phosphorylation of GSK3β were seemly not affected by ERs silencing in PC9/G cells (Fig. 3a). It indicated that ERs could inhibit β-Catenin degradation by inducing GSK3β phosphorylation. However, ERs might also bypass GSK3β to regulate the expression of β-Catenin.

To verify the effect of ERs silencing on Notch1 and GSK3β/β-Catenin pathways, 17β-E2 and Fulvestrant, the stimulant and inhibitor of ERs, respectively, were used to treat PC9/G cells. After treated with 17β-E2, ERα and ERβ expression was increased (Fig. 3c, E2 group). Consistent with elevated ERs expression, the expression of Notch1, NICD and Hes1 was also increased (Fig. 3d, E2 group). For GSK3β/β-Catenin signal, β-Catenin expression was slightly elevated, while the expression of GSK3β was not changed in PC9/G cells (Fig. 3e, E2 group). The effects of Fulvestrant on ERs, Notch1 and GSK3β/β-Catenin pathways were opposite to that of 17β-E2. After treated with Fulvestrant, the expression of ERα, ERβ, Notch1, NICD, Hes1 and β-Catenin was significantly decreased (Fig. 3c–e, Ful group). The results suggested that fluctuations in ERs expression could affect the expression or activation of key members in Notch1 pathway and affect the accumulation of β-Catenin.

### Computational modeling illustrated the dynamic process of the integrated signaling network

Combined with the information reported in [27–30] and the results in this study, a kinetic model was constructed to illustrate the dynamic process of the integrated signaling network (Additional file 5: Fig. S3). For simplicity, we made the following assumptions: ERK/SOS feedback was removed because ERK was continuously activated after EGF stimulation (Additional file 6: Fig. S4B), the overactivation of EGFR, ERs and Notch1 pathways was initiated by high amount of EGF, 17β-E2 and Dll1, respectively; pAkt, pERK, β-Catenin and Hes1 were regarded as the output indicators of the model; cell proliferation, apoptosis and mobility-associated molecules were regulated by these model outputs and then affected cell behaviors. The time-dependent expression data of key proteins (Additional file 6: Fig. S4) were used to estimate and modify model parameters. Dynamic variables, rate equations and parameters of the model could be found in Additional file 7: Tables S3–S5.

**Fig. 4:**
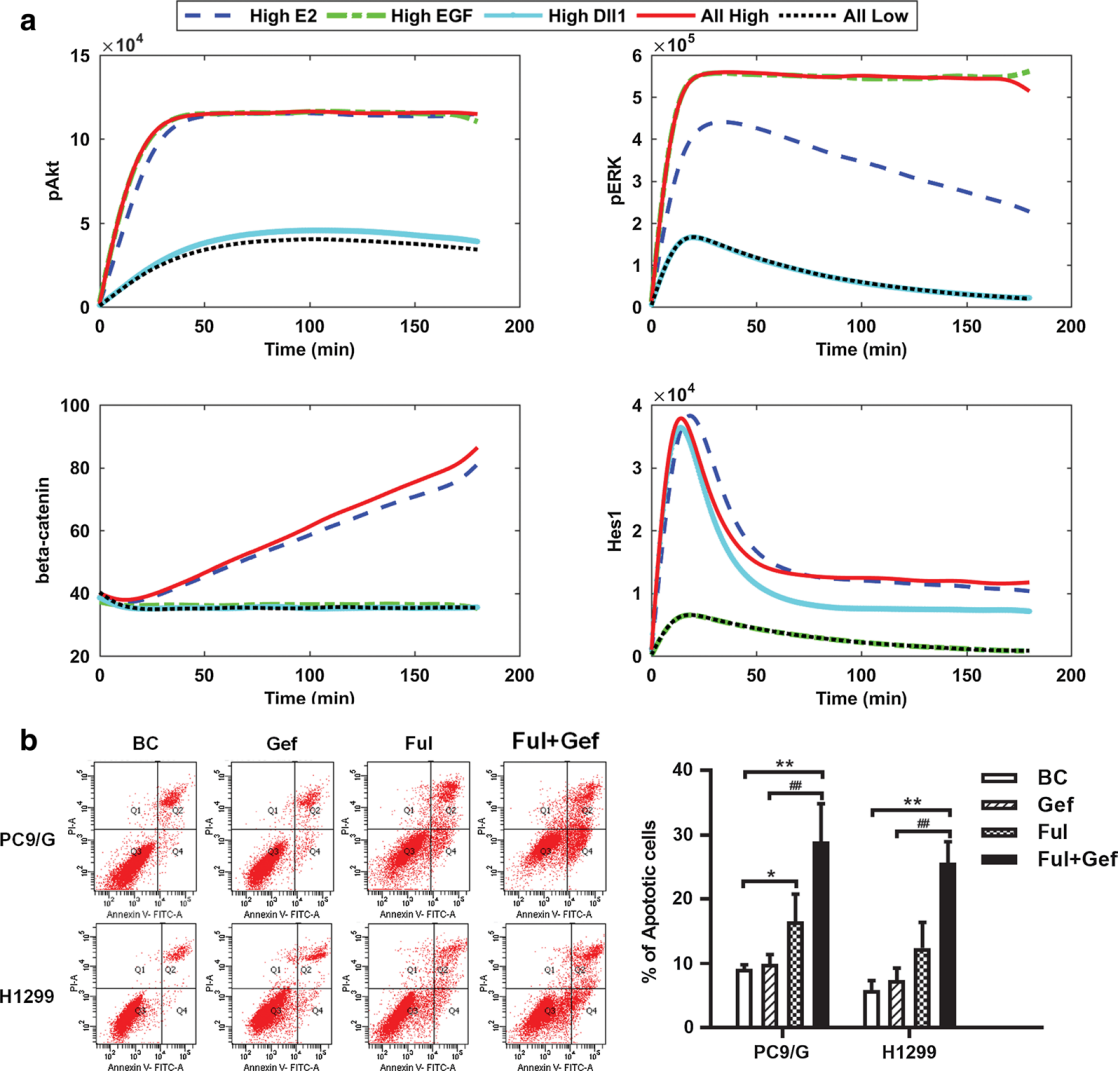
The effects of different network status on the output indicators and cell apoptosis. **a** The effects of different stimulations on the output indicators. EGFR, Notch1, ERs were supposed to be activated by high amount of EGF, Dll1 and 17β-E2, respectively. The relative amounts of output indicators pAkt, pERK, β-Catenin and Hes1 were monitored. **b** The effects of combination treatment of Gefitinib (20 μM) and Fulvestrant (1 μM) for 48 h on cell apoptosis. Cell apoptosis amount was determined by flow cytometry analysis. All experiments were repeated at least three times. *p*-values vs. Blank Control (BC) were estimated using two-tailed unpaired Student’s t-test, **p < 0.01. *p*-values vs. Gefitinib group (Gef) were estimated using two-tailed unpaired Student’s t-test, ^##^*p *< 0.01

The effects of overactivation of different pathways on the output indicators were shown in Fig. 4a. When EGF, 17β-E2 and Dll1 were all at low levels (All-Low group), the network was in an inactive state, and the outputs were maintained at low levels. When EGFR pathway was activated (High-EGF group), Akt and ERK were highly phosphorylated, but β-Catenin and Hes1 were not significantly affected. When Notch1 pathway was activated (High-Dll1 group), rapid and transient activation of Hes1 appeared, and the pAkt level was slightly elevated due to the Hes1/PTEN/PIP3 signaling. 17β-E2 (High-E2 group) could directly activate EGFR, then caused high activation of Akt and ERK. Moreover, the expression of β-Catenin and Hes1 was elevated by increased ERs expression and activation. If all pathways were activated (All-High group), the four output indicators would reach the highest levels. These results indicated that ERs activation did not show significant advantage when EGFR and Notch1 had been both highly activated, but if the two pathways were inhibited, ERs would reactivate the signaling network and induce high-level outputs.

According to the modeling result, it could be predicted that even if EGFR pathway was inhibited, hyperactivation of ERs would reactive the EGFR pathway (High-E2 group in Fig. 4a). This prediction was confirmed by our experiments: the combination treatment of Gefitinib and Fulvestrant to NSCLC cells showed better tumor suppression effects (Fig. 4b); for Gefitinib-resistant PC9/G and H1299 cells, ERs silencing could also enhance the tumor suppression effects of Gefitinib (Fig. 2a–c and Additional file 4: Fig. S2A–C). It indicated potential strategies for overcoming drug resistance of NSCLC.

### High ERα, ERβ, EGFR and Notch1 expression correlated with poor prognosis of advanced NSCLC

**Fig. 5:**
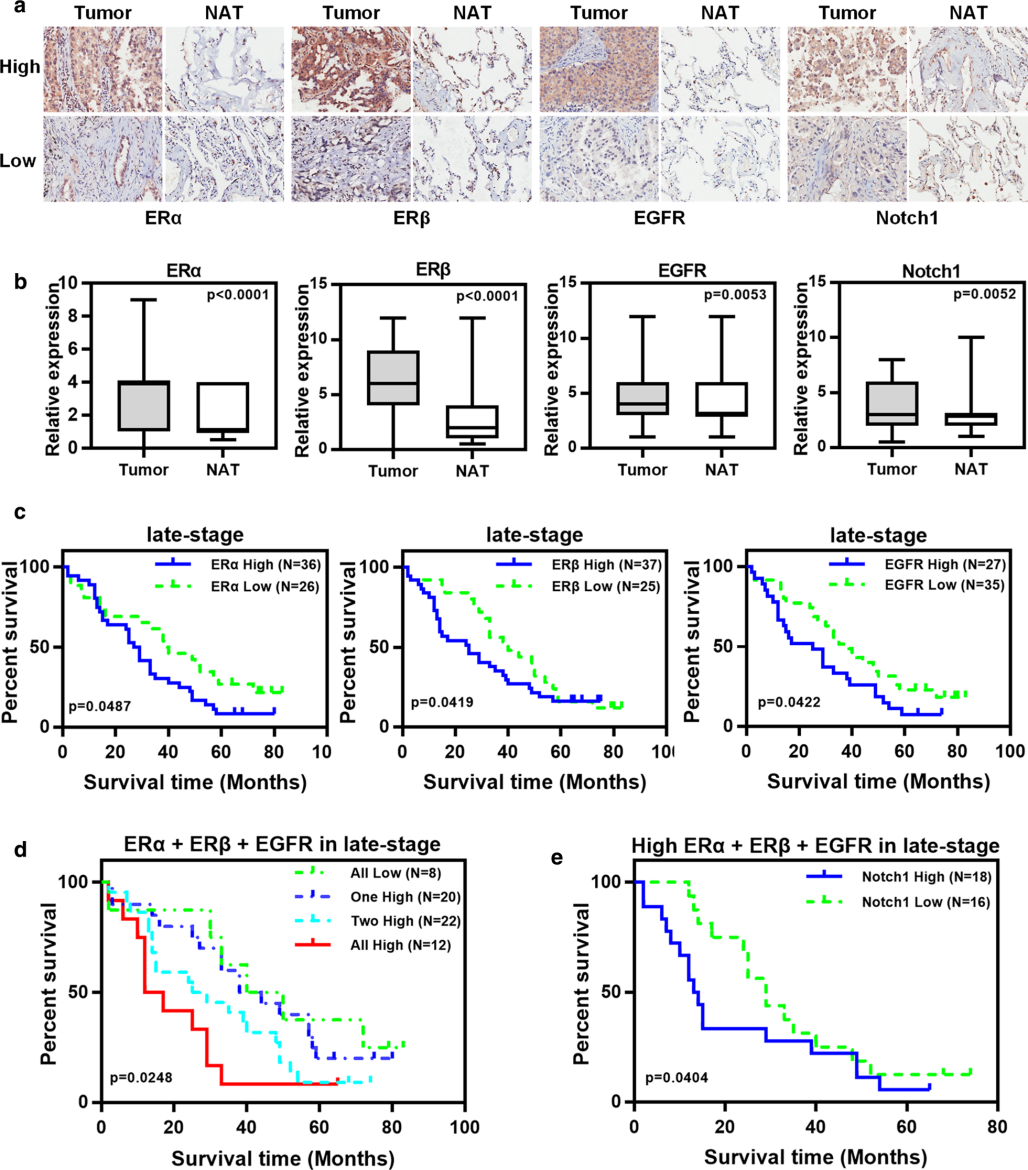
High ERs, EGFR and Notch1 expression correlated with poor prognosis of advanced NSCLC. **a** Representative examples of IHC expression of ERα, ERβ, EGFR and Notch1 in NSCLC tumor tissues (Tumor) and the paired non-tumor adjacent tissues (NAT). Original magnification: ×200 for all samples. **b** Unpaired *t* test of ERα, ERβ, EGFR and Notch1 between Tumor group (N = 93) and NAT group (N = 87). IHC score was an index of ERα, ERβ, EGFR and Notch1 expression levels. **c** Kaplan–Meier curves showing overall survival of the late-stage NSCLC patients for ERα, ERβ and EGFR expression, respectively. **d** Overall survival of the late-stage NSCLC patients with grouped high-expression receptors. **e** Overall survival of the late-stage NSCLC patients with at least two high-expression receptors for Notch1 expression. *p* values were calculated using the log-rank test

Here we also evaluated the clinical relevance of these four membrane receptors ERα, ERβ, EGFR and Notch1 in NSCLC patients. Demographic information of all patients was shown in Additional file 8: Table S6. IHC results showed that the expressions of ERα, ERβ, EGFR and Notch1 in tumor tissues were higher than those in adjacent normal tissues (Fig. 5a, b). However, there were no significant results in the Kaplan–Meier analysis for correlations between the expression levels of each receptor and survival outcomes (data not shown). Considering that the degree of malignancy served as one of the most important predictive factors for NSCLC prognosis, the samples were divided into early-stage group (stage I) and late-stage group (stage II–IV). Survival analysis demonstrated that high levels of ERα, ERβ, or EGFR were significantly correlated with worse 10-year overall survival for the late-stage NSCLC patients (Fig. 5c). But for the early-stage NSCLC patients, there was still no significant results in survival analysis (data not shown). For the late-stage NSCLC group, the survival outcomes of patients with grouped high-expression receptors were further analyzed. As shown in Fig. 5d, the more receptors that were highly expressed, the worse the patients’ survival were. Moreover, in the subgroup with at least two high-expression receptors in Fig. 5d, patients with high-expression Notch1 displayed worse survival outcomes (Fig. 5e). These results suggested that ERs, EGFR and Notch1 were poor prognostic factors for advanced NSCLC, and that these receptors had a synergistic effect on poor prognosis of advanced NSCLC.

## Discussion

Over the past few decades, many reports have proposed that ERs play an important role in NSCLC [1–3]. However, the effect of ERs in NSCLC is still controversial. The mechanisms of ERs in NSCLC are also not clear enough. So this study, we reconsidered the role of ERs in NSCLC from the perspective of cancer systems biology. And we suggested that ERs promoted NSCLC progression through modulating the integrated membrane receptor signaling network rather than individual targets to maintain and enhance the tumor cell behaviors. These results also indicated that during the evolution of cancer such as NSCLC, various carcinogenic factors interact with each other to maintain tumor phenotypes. This opinion was also supported by other researchers [31, 32].

To gain a preliminary understanding of ERs in NSCLC, effects of ESR1/2 expression fluctuations on the expressions and functions of the genome in NSCLC patients were first analyzed. GO and KEGG analysis showed that activation and transduction of the membrane receptor signaling pathways were likely to be affected by ESR1/2 variation. DEGs were involved the growth factor signaling pathway, Wnt/GSK/β-Catenin pathway and Notch pathway. These pathways had been reported to play an vital role in the development and progression of NSCLC. As the important therapeutic targets of NSCLC, EGFR [33], fibroblast growth factor receptor (FGFR) [34] and type 1 insulin-like growth factor receptor (IGF1R) [35] pathways were also reported to crosstalk with ERs signals [8, 36, 37]. It was also reported that Notch [38] and Wnt/β-Catenin [39] pathways could participate in NSCLC progression. However, the relationship between ERs and Notch, Wnt/β-Catenin pathways in NSCLC was rarely discussed. Therefore, we subsequently investigated the effects of ERs on key proteins of Notch1 and GSK3β/β-Catenin pathways and on the membrane receptor signal network composed of EGFR, Notch1 and GSK3β/β-Catenin pathways.

In addition to the membrane receptor signaling pathways, DEGs were also involved in the following genes: complement system members (C3, C7, etc.), chemokines and their receptors (CCL13, CCR2, CX3CR1, CXCL2, etc.), CD molecules (CD2, CD4, CD22, etc.), tumor necrosis factor receptor superfamily (TNFRSF10C, TNFSF8, etc.), cadherin related members (CDH8, CDHR1, CDHR4, etc.) and so on. All of these terms could be attributed to the tumor microenvironment, suggesting that regulating the integrated balance of tumor microenvironment might also be one of the potential mechanisms of ERs in NSCLC [40–43]. Since the tumor microenvironment is not the focus of this study, we plan to incorporate immune-related pathways, especially chemokine receptors, into the signal network model in our future research.

As shown in the results of bioinformatics analysis, ESR1/2 might regulate many genes and pathways such as EGFR, Notch and Wnt/β-Catenin pathways, most of which were involved in the development and progression of NSCLC. Activation of EGFR could lead to autophosphorylation of receptor tyrosine kinase and subsequent regulate cell proliferation, differentiation and survival [44]. Notch and β-Catenin signals were proved to be important promoting factors of NSCLC metastasis [45, 46]. Therefore, ERs might promote NSCLC cell proliferation, migration, invasion and apoptosis escape by regulating these pathways. Before investigating the molecular mechanisms of ERs in NSCLC, we first investigated the influence of ERs in NSCLC at cellular level by detecting the effects of ERs on NSCLC cell phenotypes.

In this study, it was emphasized that ERs could promote cell migration and invasion by regulating EMT. The expression of epithelial and mesenchymal markers was regulated by ERs (Fig. 2e and Additional file 4: Fig. S2E). Among them, the key markers E-Cadherin and N-Cadherin were important cell adhesion molecules. Cell adhesion was also significant enriched in GO and KEGG analysis, which is the molecular basis of various physiological and pathological processes such as signaling transduction, cell differentiation, invasion and migration [47]. EMT reduced the adhesion capacity of tumor cells and lead to cell invasion and migration, which might eventually cause tumor metastasis [48]. Besides tumor metastasis, Hamilton et al. also reported that ERα could induce chemotherapy resistance by promoting EMT [49].

Apoptosis escape was one of the reasons for uncontrolled growth of tumor cells, which in turn leads to continuous evolution of tumors [50]. Promoting apoptosis has been considered as an effective strategy for oncotherapy [51]. We found that ERs could inhibit cell apoptosis by regulating the expression of anti- and pro-apoptotic proteins to inhibit cell apoptosis. In addition, ERs inhibition combined with EGFR tyrosine kinase inhibitor resistance (TKI) such as Gefitinib could increase the amount of cell apoptosis, consistent with the results of Stabile et al. [8, 52]. Another research reported that ERβ from the mitochondrial fraction could exert its apoptosis-inhibition function by disrupting Bad-Bcl-XL and Bad-Bcl-2 interactions [53]. Besides, GO analysis showed that calcium ion binding was a significant enrichment term (Tables 1, 2 and Additional file 2). Some researchers pointed out that pro- and anti-apoptotic proteins regulated the intracellular calcium homeostasis, which could affect the efficiency of various apoptosis inducing agents. And this regulation process was ER-associated [54]. Anyhow, it was consistent with the opinion that ERs regulated the balance between pro- and anti-apoptotic proteins through multiple signals.

In our research, ERs were suggested to regulate the expression and activation of key proteins in EGFR, Notch1 and GSK3β/β-Catenin pathways (Fig. 3 and Additional file 6: Fig. S4). The results also suggested that ERs modulated the signaling network through the following nodes: phosphorylate EGFR; phosphorylate GSK3β by pAkt/pERK and then reduce the phosphorylation and degradation of β-catenin; upregulated β-catenin expression bypass GSK3β signal; increase Notch1 expression and activate NICD signal. That is, ERs modulated the signaling network by adding redundant and positive-feedback paths to maintain the stable outputs of carcinogenic signals, and then to promote tumor phenotypic stability and tumor progression. The redundancy and feedback effects of signaling networks were main reasons for the complexity and refractory of cancers, which had been widely discussed [55, 56]. It was also one of the important reasons for acquired resistance of molecular targeted drugs [57]. Our results confirmed that ERs lead to NSCLC resistance by reactivate the redundant pathways in the signaling network. Targeting ERs could alleviate EGFR TKI had been reported by Stabile et al. [8, 52], which suggests potential strategies for overcoming drug resistance of NSCLC.

Our modeling results suggested that these membrane receptor pathways constituted an integrated network to cooperatively promote NSCLC progression. The IHC results echoed that the four membrane receptors ERα, ERβ, EGFR and Notch1 had a synergistic effect on poor prognostic effect of advanced NSCLC. But for the early-stage NSCLC, no significant results were observed. These results were supported by the idea that mutations and abnormal expressions of the genome in the advanced stage of a cancer were much more frequent than those in the early stage.

It should be noted that this study has some limitations. Because of some assumptions made for simplification, our model should be considered as an approximate description rather than an exact definition of the signaling network. In addition, several pathways such as EGFR and Notch1 were involved to illustrate the regulation of ER on molecular networks in our research. However, signals involved in tumor progression of NSCLC go far beyond those. We provided a research prototype here, and we hoped to gradually refine the signaling network model of NSCLC in the future.

## Conclusions

In summary, this study aimed to explore the tumor-promoted mechanism of ERs in NSCLC from the perspective of cancer systems biology. ERs might affect many cancer-related molecular events and pathways in NSCLC, particularly the membrane receptor signaling pathways, which might ultimately lead to changes in cell behaviors. The promotive effect of ERs in NSCLC progression was achieved by modulating the signaling network composed of EGFR, Notch1, GSK3β/β-Catenin pathways, and then regulating cell proliferation, mobility and apoptosis. IHC analysis echoed that ERs, EGFR and Notch1 had a synergistic effect on poor prognosis of advanced NSCLC. Overall, this study suggested that ERs were likely to facilitate NSCLC progression by modulating the integrated signaling network and maintaining the stable outputs of oncogenic signals. It also complemented the molecular mechanisms underlying the progression of NSCLC and provided new opportunities for optimizing therapeutic scheme and for improving clinical outcomes in NSCLC. On the other hand, this study encouraged systemic and comprehensive perspectives of cancer and had made some new attempts at the methodology of cancer research.

## Supplementary information

**Additional file 1: Table S1–S2.** Antibody information for Western blot analysis and immunohistochemical analysis.

**Additional file 2.** Complete results of the GO and KEGG enrichment analysis.

**Additional file 3: Fig. S1.** The effect of ERs on cell proliferation after 17β-E2 stimulation and the expression of ERα and ERβ in NSCLC cells.

**Additional file 4: Fig. S2.** The effects of ERs silencing on H1299 cell migration, invasion and apoptosis.

**Additional file 5. Fig. S3.** The schematic diagram of molecular signaling network in NSCLC.

**Additional file 6: Fig. S4.** The expression levels of key molecules in Notch1, EGFR, ERs and GSK3β/β-Catenin pathways after stimulated by Dll1, EGF and 17β-E2, respectively.

**Additional file 7: Table S3–S5.** Information of computational model of the signaling network.

**Additional file 8: Table S6.** Patient cohort characteristics of all patients (N = 93).

## Data Availability

The TCGA dataset (ID: TCGA.LUNG.sampleMap/HiSeqV2) was free to download from the UCSC Xena (https://xenabrowser.net/datapages/). Other data generated or analyzed during this study were included in this published article and its additional files.

## Abbreviations

DAVID: The Database for Annotation, Visualization and Integrated Discovery online tool
DEGs: differently expressed genes
EGFR: epidermal growth factor receptor
EMT: epithelial–mesenchymal transition
ER: estrogen receptor
FGFR: fibroblast growth factor receptor
GO: Gene Ontology
GSK3β: glycogen synthase kinase-3β
IGF1R: type 1 insulin-like growth factor receptor (IGF1R)
IHC: immunohistochemistry
KEGG: Kyoto Encyclopedia of Genes and Genomes
NICD: intracellular domain of Notch1 receptor
NSCLC: non-small cell lung cancer
TKI: tyrosine kinase inhibitor
