# Supplementary material for: Estrogen receptors promote NSCLC progression by modulating the membrane receptor signaling network: a systems biology perspective
Source: J Transl Med. 2019 Sep 11;17:308. doi: 10.1186/s12967-019-2056-3 (PMC6737693; doi:10.1186/s12967-019-2056-3)
Supplement: Supplementary file 1 — Additional file 1: Table S1–S2. Antibody information for Western blot analysis and immunohistochemical analysis. [file 12967_2019_2056_MOESM1_ESM.doc]

# **Additional file 1**

**Table S1** Antibody information for Western blot analysis

| **Antibody** | **Manufacturer** | **Catalog** | **Dilution** |
| --- | --- | --- | --- |
| ERα | Affinity | DF6094 | 1: 1000 |
| ERβ | Affinity | AF6469 | 1: 1000 |
| EGFR | Abbkine | 51071-2-AP | 1: 1000 |
| pEGFR | Abcam | Ab32578 | 1: 1000 |
| Akt | CST | 4691T | 1: 1000 |
| pAkt | Affinity | AF0908 | 1: 1000 |
| ERK | Abcam | Ab184699 | 1: 10000 |
| pERK | Affinity | AF1015 | 1: 1000 |
| Notch1 (C-20) | Santa Cruz | sc-6014 | 1: 200 |
| NICD | Affinity | AF5307 | 1: 1000 |
| Hes1 | Ab108937 | Abcam | 1: 1000 |
| PTEN | Affinity | AF6351 | 1: 1000 |
| GSK3β | CST | 12456T | 1: 1000 |
| pGSK3β | Abcam | Ab75814 | 1: 10000 |
| β-catenin | Santa Cruz | Sc-7199 | 1: 400 |
| E-Cadherin | CST | 3195P | 1: 1000 |
| N-Cadherin | CST | 13116 | 1: 1000 |
| Fibronectin | Abcam | ab32419 | 1: 1000 |
| ZEB1 | Abcam | ab203829 | 1: 1000 |
| Snail | CST | 3879P | 1: 1000 |
| Vimentin | CST | 3932S | 1: 1000 |
| Survivin | CST | 2808T | 1: 1000 |
| Bim | CST | 2933T | 1: 1000 |
| Bcl-2 | CST | 15071T | 1: 1000 |
| Cleaved Caspase3 | CST | 9664T | 1: 1000 |
| GAPDH | Abbkine | 60004-Hg | 1: 10000 |
| β-Actin | Abbkine | A01011-1 | 1: 5000 |
| β-Tubulin | Abbkine | 10094-1-AP | 1: 5000 |

**Table S2** Antibody information for immunohistochemical analysis

| **Antibody** | **Manufacturer** | **Catalog** | **Dilution** |
| --- | --- | --- | --- |
| ERα (HC-20) | Santa Cruz | sc-543 | 1: 160 |
| ERβ (B-1) | Santa Cruz | sc-390243 | 1: 500 |
| EGFR | Abbkine | 51071-2-AP | 1: 50 |
| Notch1 (C-20) | Santa Cruz | sc-6014 | 1: 200 |
