# Supplementary figures and images for: Estrogen receptors promote NSCLC progression by modulating the membrane receptor signaling network: a systems biology perspective
Source: J Transl Med. 2019 Sep 11;17:308. doi: 10.1186/s12967-019-2056-3 (PMC6737693; doi:10.1186/s12967-019-2056-3)

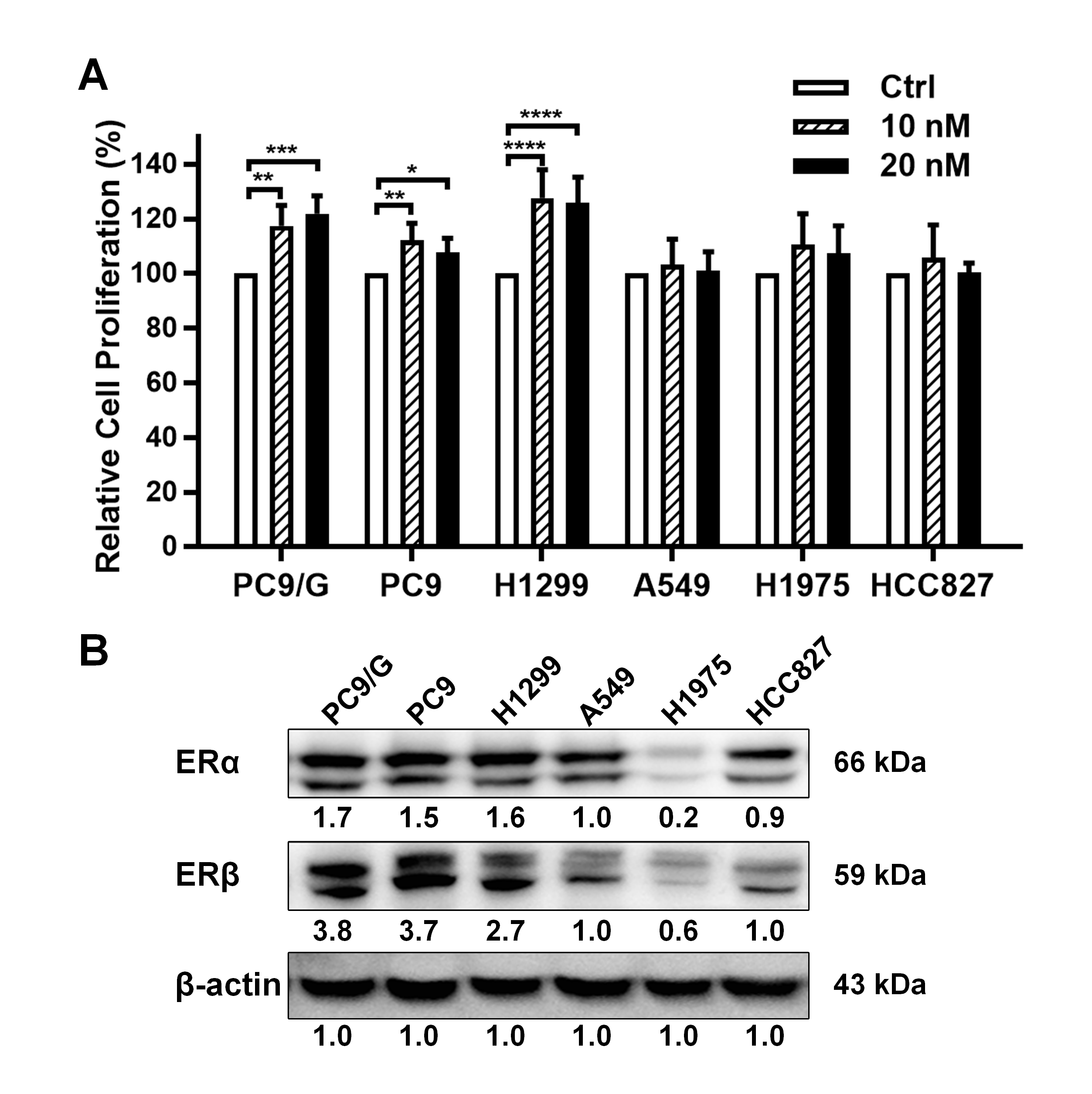

Supplement: Supplementary file 3 — Additional file 3: Fig. S1. The effect of ERs on cell proliferation after 17β-E2 stimulation and the expression of ERα and ERβ in NSCLC cells. [file 12967_2019_2056_MOESM3_ESM.tif]

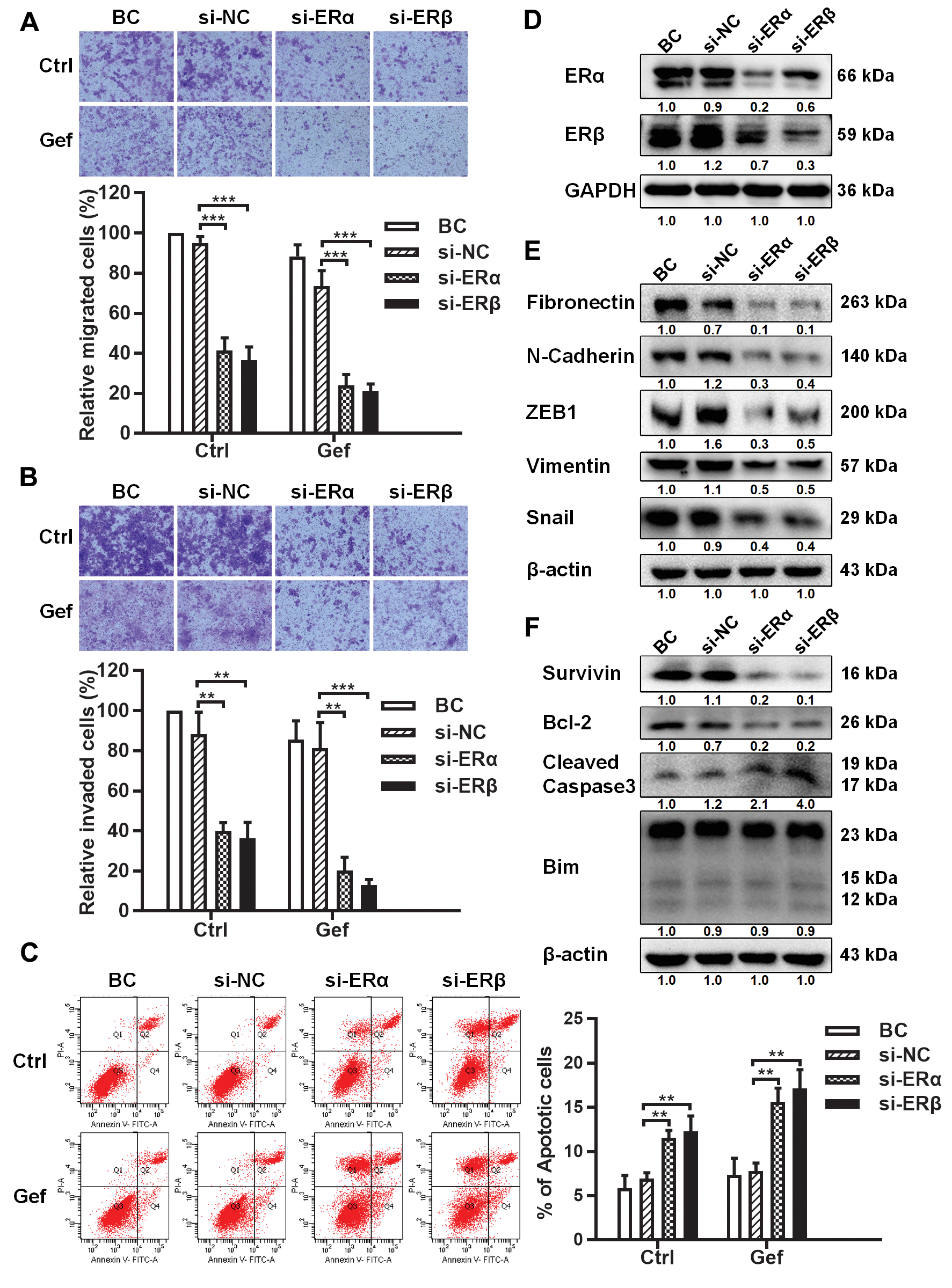

Supplement: Supplementary file 4 — Additional file 4: Fig. S2. The effects of ERs silencing on H1299 cell migration, invasion and apoptosis. [file 12967_2019_2056_MOESM4_ESM.tif]

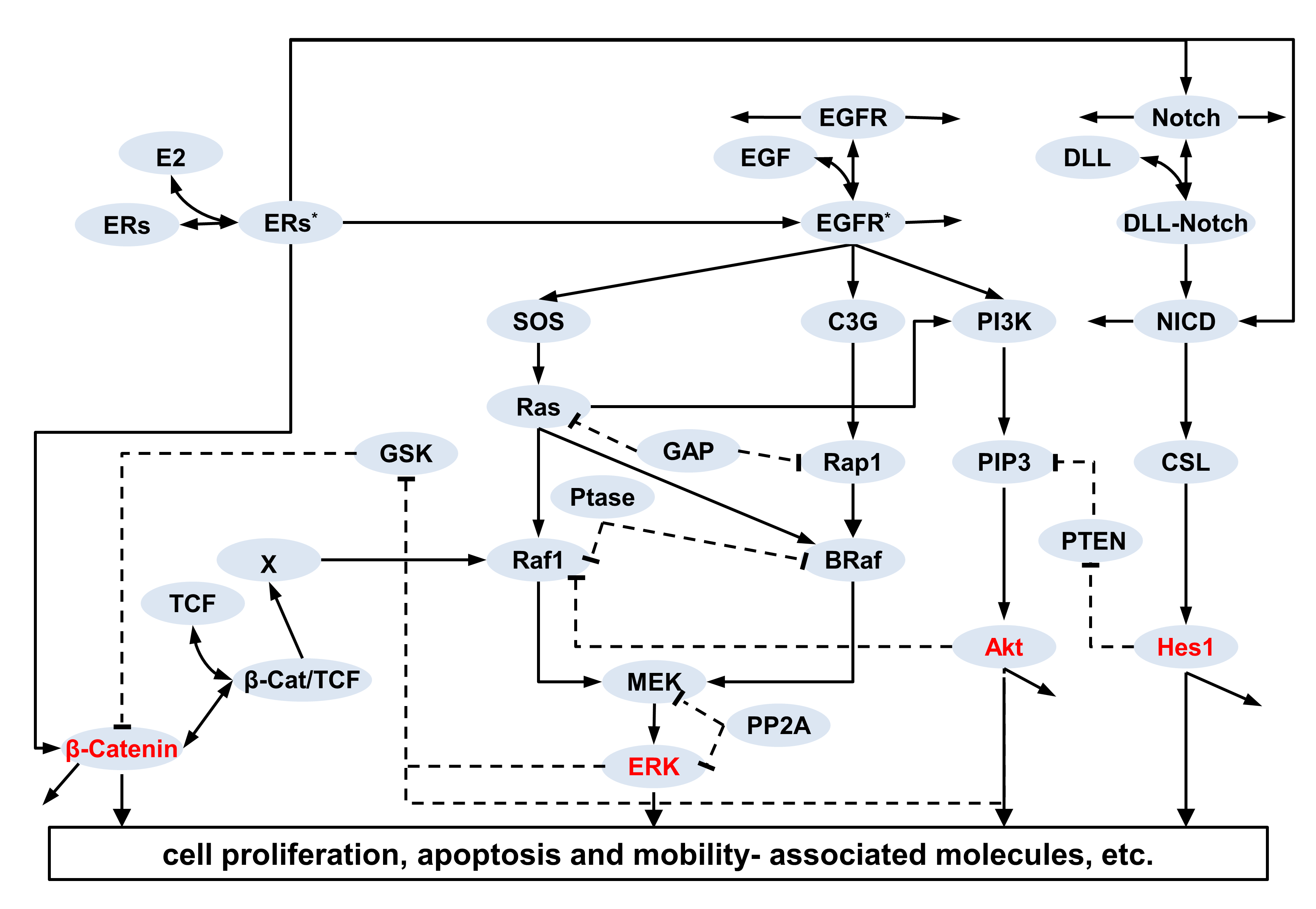

Supplement: Supplementary file 5 — Additional file 5. Fig. S3. The schematic diagram of molecular signaling network in NSCLC. [file 12967_2019_2056_MOESM5_ESM.tif]

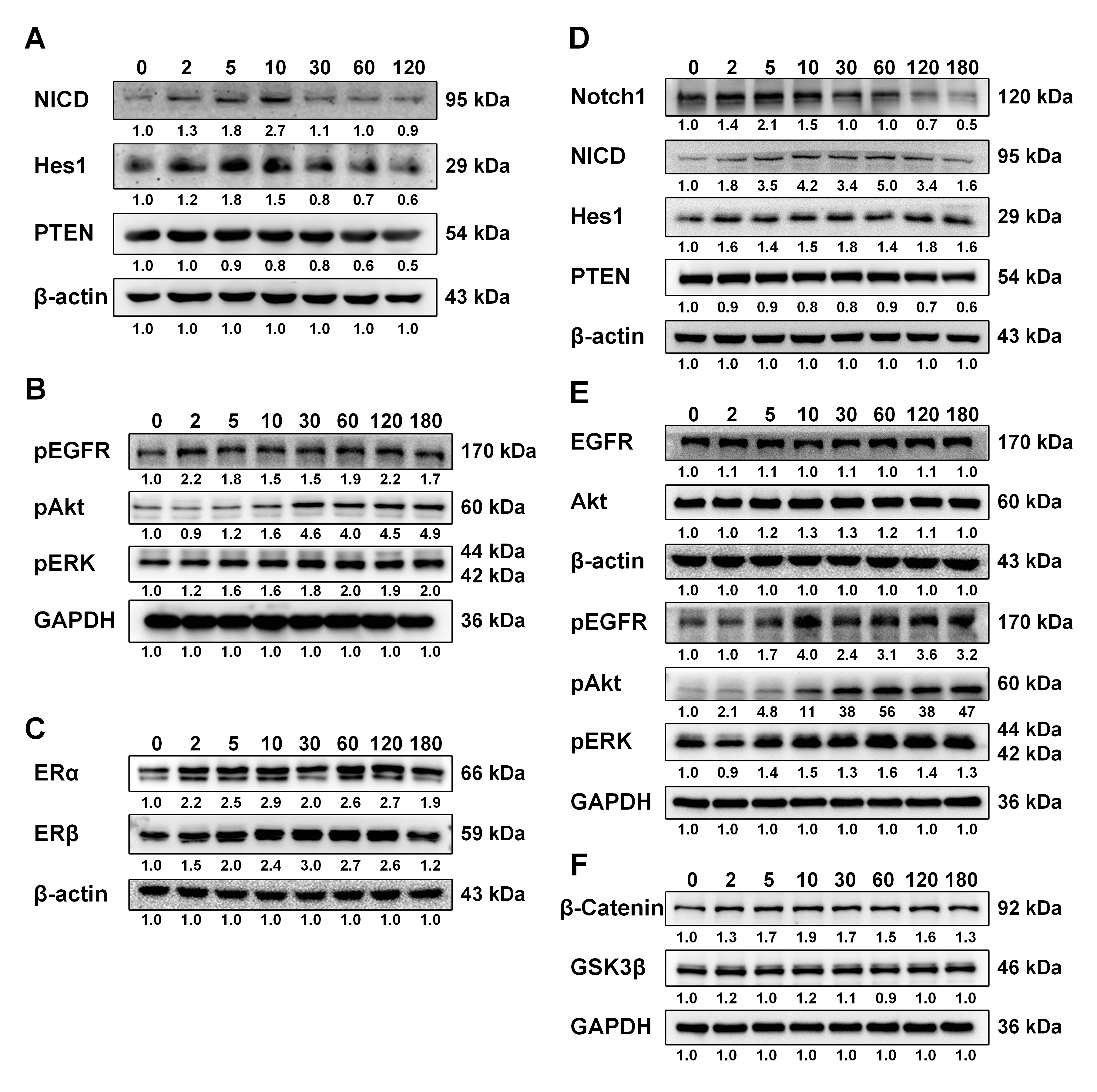

Supplement: Supplementary file 6 — Additional file 6: Fig. S4. The expression levels of key molecules in Notch1, EGFR, ERs and GSK3β/β-Catenin pathways after stimulated by Dll1, EGF and 17β-E2, respectively. [file 12967_2019_2056_MOESM6_ESM.tif]
