# Supplementary material for: Estrogen receptors promote NSCLC progression by modulating the membrane receptor signaling network: a systems biology perspective
Source: J Transl Med. 2019 Sep 11;17:308. doi: 10.1186/s12967-019-2056-3 (PMC6737693; doi:10.1186/s12967-019-2056-3)
Supplement: Supplementary file 7 — Additional file 7: Table S3–S5. Information of computational model of the signaling network. [file 12967_2019_2056_MOESM7_ESM.doc]

# **Additional file 7**

**Table S3** Ordinary Differential Equations used to construct the mathematical model of the molecular signaling network.

| **ODEs:** |
| --- |
| d([EGF])/dt = -V1+V2 |
| d([EGFR])/dt = -V1+V2+V3-V4-V60+V61 |
| d([EGFR*])/dt = V1-V2-V5 |
| d([SOS])/dt = -V6+V7-V63 |
| d([SOS*])/dt = V6-V7+V63 |
| d([Ras])/dt = -V8+V9 |
| d([Ras*])/dt = V8-V9 |
| d([Raf1])/dt = -V10+V11+V12-V51 |
| d([Raf1*])/dt = V10-V11-V12+V51 |
| d([C3G])/dt = -V13+V14-V64 |
| d([C3G*])/dt = V13-V14+V64 |
| d([Rap1])/dt = -V15+V16 |
| d([Rap1*])/dt = V15-V16 |
| d([BRaf])/dt = -V17-V18+V19 |
| d([BRaf*])/dt = V17+V18-V19 |
| d([MEK])/dt = -V20 - V21 + V22 |
| d([MEK*])/dt = V20+V21-V22 |
| d([ERK])/dt = -V23+V24 |
| d([ERK*])/dt = V23-V24 |
| d([PI3K])/dt = -V25-V26+V27-V65 |
| d([PI3K*])/dt = V25+V26-V27+V65 |
| d([PIP3])/dt = V28-V29 |
| d([PIP2])/dt = -V28+V29 |
| d([Akt])/dt = -V30+V31 |
| d([Akt*])/dt = V30-V31 |
| d([Dll1])/dt = -V32+V33 |
| d([Notch1])/dt = -V32+V33-V34+V35+V58-V59 |
| d([Notch1-TM])/dt = V32-V33-V36 |
| d([NICD])/dt = V36-V37+V59 |
| d([RBPJ])/dt = -V38+V39 |
| d([RBPJ*])/dt = V38-V39 |
| d([Hes1])/dt = V40-V41 |
| d([PTEN])/dt = -V4) |
| d([GSK3B])/dt = -V43-V44+V45 |
| d([pGSK3B])/dt = V43+V44-V45 |
| d([beta-Catenin])/dt = -V46-V47+V48+V66 |
| d([TCF])/dt = -V47+V48 |
| d([TCF_beta-Catenin])/dt = V47-V48 |
| d([X])/dt = V49-V50 |
| d([E2])/dt = -V52+V53-V60+V61) |
| d([ER])/dt = -V52+V53+V54+V55-V56 |
| d([ER*])/dt = V52-V53-V57 |
| d([EGFR_E2])/dt = V60-V61-V62 |
| **Fluxes:** |
| V1 = K1×[EGF] ×[EGFR] |
| V2 = K2×[EGFR*] |
| V3 = K3 |
| V4 = K4×[EGFR] |
| V5 = K5×[EGFR*] |
| V6 = K6×[EGFR*]×[SOS]/(K7+[SOS]) |
| V7 = K8×[SOS*] |
| V8 = K15×[SOS*]×[Ras]/(K16+[Ras]) |
| V9 = K17×[RasGap] ×[Ras*]/(K18+[Ras*]) |
| V10 = K19×[Ras*]×[Raf1]/(K20+[Raf1]) |
| V11 = K21×[Akt*]×[Raf1*]/(K22+[Raf1*]) |
| V12 = K23×[Ptase] ×[Raf1*]/(K24+[Raf1*]) |
| V13 = K9×[EGFR*]×[C3G]/(K10+[C3G]) |
| V14 = K11×[C3G*] |
| V15 = K25×[C3G*]×[Rap1]/(K26+[Rap1]) |
| V16 = K27×[Rap1Gap] ×[Rap1*]/(K28+[Rap1*]) |
| V17 = K29×[Rap1*]×[BRaf]/(K30+[BRaf]) |
| V18 = K31×[Ras*]×[BRaf]/(K32+[BRaf]) |
| V19 = K33×[Ptase] ×[BRaf*]/(K34+[BRaf*]) |
| V20 = K35×[Raf1*]×[MEK]/(K36+[MEK]) |
| V21 = K37×[BRaf*]×[MEK]/(K38+[MEK]) |
| V22 = K39×[PP2A] ×[MEK*]/(K40+[MEK*]) |
| V23 = K41×[MEK*]×[ERK]/(K42+[ERK]) |
| V24 = K43×[PP2A] ×[ERK*]/(K44+[ERK*]) |
| V25 = K45×[EGFR*]×[PI3K]/(K46+[PI3K]) |
| V26 = K12×[Ras*]×[PI3K]/(K13+[PI3K]) |
| V27 = K14×[PI3K*] |
| V28 = K47×[PI3K*]×[PIP2]/(K48+[PIP2]) |
| V29 = K49×[PTEN] ×[PIP3]/(K50+[PIP3]) |
| V30 = K51×[PIP3] ×[Akt]/(K52+[Akt]) |
| V31 = K53×[Akt*] |
| V32 = K54×[Notch1][Dll1] |
| V33 = K55×[Notch1-TM] |
| V34 = K56×[Notch1] |
| V35 = K57 |
| V36 = K58×[Enzyme] ×[Notch1-TM]/(K59+[Notch1-TM]) |
| V37 = K60×[NICD] |
| V38 = K61×[NICD] ×[RBPJ]/(K62+[RBPJ]) |
| V39 = K63×[RBPJ*] |
| V40 = K64×[RBPJ*] |
| V41 = K65×[Hes1] |
| V42 = K66×[Hes1] ×[PTEN]/(K67+[PTEN]) |
| V43 = K68×[GSK3B] ×[Akt*] |
| V44 = K69×[GSK3B] ×[ERK*] |
| V45 = K70×[pGSK3B] |
| V46 = K71×[GSK3B] ×[beta-Catenin]/(K72+[beta-Catenin]) |
| V47 = K73×[TCF] ×[beta-Catenin] |
| V48 = K74×[TCF_beta-Catenin] |
| V49 = K75×[TCF_beta-Catenin] ^2/(K76^2+[TCF_beta-Catenin]^2) |
| V50 = K77×[X] |
| V51 = K78×[X] ×[Raf1]/(K79+[Raf1]) |
| V52 = K80×[E2] ×[ER] |
| V53 = K81×[ER*] |
| V54 = K82 |
| V55 = K83×[E2] |
| V56 = K84×[ER] |
| V57 = K85×[ER*] |
| V58 = K86×[ER*] |
| V59 = K87×[ER*]×[Notch1]/(K88+[Notch1]) |
| V60 = K89×[EGFR] ×[E2] |
| V61 = K90×[EGFR_E2] |
| V62 = K91×[EGFR_E2] |
| V63 = K92×[EGFR_E2] ×[SOS]/(K93+[SOS]) |
| V64 = K94×[EGFR_E2] ×[C3G]/(K95+[C3G]) |
| V65 = K96×[EGFR_E2] ×[PI3K]/(K97+[PI3K]) |
| V66 = K98×[ER*] |

**Table S4** Parameter values used for simulation of the molecular signaling network

| **Parameter** | **Value** | **Reference** |
| --- | --- | --- |
| K1 | 2.185E-05 |  |
| K2 | 0.012 |  |
| K3 | 1000 | Fix |
| K4 | 0.00125 |  |
| K5 | 0.0146713 | Estimated |
| K6 | 694.731 |  |
| K7 | 6086070 |  |
| K8 | 2.5 |  |
| K9 | 96.907410 | Estimated |
| K10 | 6262805.7 | Estimated |
| K11 | 2.5 |  |
| K12 | 0.0771067 |  |
| K13 | 272056 |  |
| K14 | 2.5 | Fix |
| K15 | 32.344 |  |
| K16 | 35954.3 |  |
| K17 | 1509.36 |  |
| K18 | 1432410 |  |
| K19 | 0.884096 |  |
| K20 | 62464.6 |  |
| K21 | 15.1212 |  |
| K22 | 119355 |  |
| K23 | 0.126329 |  |
| K24 | 1061.71 |  |
| K25 | 32.344 |  |
| K26 | 35954.3 |  |
| K27 | 1509.36 |  |
| K28 | 1432410 |  |
| K29 | 0.884096 |  |
| K30 | 62464.6 |  |
| K31 | 0.884096 |  |
| K32 | 62464.6 |  |
| K33 | 0.126329 |  |
| K34 | 1061.71 |  |
| K35 | 185.759 |  |
| K36 | 4768350 |  |
| K37 | 185.759 |  |
| K38 | 4768350 |  |
| K39 | 2.83243 |  |
| K40 | 518753 |  |
| K41 | 9.85367 |  |
| K42 | 1007340 |  |
| K43 | 8.8912 |  |
| K44 | 3496490 |  |
| K45 | 10.6737 |  |
| K46 | 184912 |  |
| K47 | 40.085331 | Estimated |
| K48 | 25968.83 | Estimated |
| K49 | 1.733496 | Estimated |
| K50 | 27019.5237 | Estimated |
| K51 | 0.1716877 | Estimated |
| K52 | 137484.0897 | Estimated |
| K53 | 0.005 |  |
| K54 | 3.885199e-05 | Estimated |
| K55 | 4.8724339 | Estimated |
| K56 | 5.8663546e-05 | Estimated |
| K57 | 500 | Fix |
| K58 | 0.2261941 | Estimated |
| K59 | 23929.0888 | Estimated |
| K60 | 0.0647534 | Estimated |
| K61 | 0.02901488 | Estimated |
| K62 | 106050.9131 | Estimated |
| K63 | 12.2279467 | Estimated |
| K64 | 1025.771912 | Estimated |
| K65 | 1.35373 | Estimated |
| K66 | 1.392612 | Estimated |
| K67 | 9699962.4213 | Estimated |
| K68 | 0.002 |  |
| K69 | 0.04596 |  |
| K70 | 0.01541 |  |
| K71 | 162.374 | Estimated |
| K72 | 74120.4 | Estimated |
| K73 | 0.01667 |  |
| K74 | 0.5 |  |
| K75 | 0.01 |  |
| K76 | 15 |  |
| K77 | 0.00025 |  |
| K78 | 0.025 |  |
| K79 | 15 |  |
| K80 | 0.00518261 | Estimated |
| K81 | 0.00410874 | Estimated |
| K82 | 300 | Fix |
| K83 | 0.00803802 | Estimated |
| K84 | 0.0272156 | Estimated |
| K85 | 0.014018 | Estimated |
| K86 | 0.0014756 | Estimated |
| K87 | 0.0637897 | Estimated |
| K88 | 9615.21 | Estimated |
| K89 | 5.84686e-08 | Estimated |
| K90 | 3.63878e-05 | Estimated |
| K91 | 0.0417503 | Estimated |
| K92 | 694.731 |  |
| K93 | 6086070 |  |
| K94 | 96.907410 | Estimated |
| K95 | 6262805.7158 | Estimated |
| K96 | 10.6737 | Estimated |
| K97 | 184912 | Estimated |
| K98 | 1.35772e-06 | Estimated |

**Table S5** Initial concentration of the species in the molecular signaling network.

| **Concentration** | **Reference** |
| --- | --- |
| EGF = 0/8000/800000 (none/low/high) | fix |
| EGFR = 80000 |  |
| EGFR* = 0 |  |
| SOS = 120000 |  |
| SOS* = 0 |  |
| Ras = 120000 |  |
| Ras* = 0 |  |
| Gap = 120000 |  |
| Raf1 = 120000 |  |
| Raf1* = 0 |  |
| Ptase = 120000 |  |
| C3G = 120000 |  |
| C3G* = 0 |  |
| Rap1 = 120000 |  |
| Rap1* = 0 |  |
| BRaf = 120000 |  |
| BRaf* = 0 |  |
| MEK = 120000 |  |
| MEK* = 0 |  |
| PP2A = 120000 |  |
| ERK = 600000 |  |
| ERK* = 0 |  |
| PI3K = 120000 |  |
| PI3K* = 0 |  |
| PIP3 = 0 |  |
| PIP2 = 120000 |  |
| Akt = 120000 |  |
| Akt* = 0 |  |
| Dll1 = 0/8000/800000 (none/low/high) | Fix |
| Notch1 = 80000 | Fix |
| Notch1-TM = 0 | Fix |
| Enzyme = 120000 | Fix |
| NICD = 0 | Fix |
| RBPJ = 120000 | Fix |
| RBPJ* =0 | Fix |
| Hes1 = 0 | Fix |
| PTEN = 600000 | Fix |
| GSK3B = 49.137 |  |
| pGSK3B = 0 |  |
| beta-Catenin =40 |  |
| TCF = 6.1879 |  |
| TCF_beta-Catenin = 0 |  |
| X = 0 |  |
| E2 = 0/8000/800000 (none/low/high) | Fix |
| ER = 80000 | Fix |
| ER* = 0 | Fix |

**Reference:**

1. Brown KS, Hill CC, Calero GA, Myers CR, Lee KH, Sethna JP, et al. The statistical mechanics of complex signaling networks: nerve growth factor signaling. Physical biology. 2004 2004/12//;1(3-4):184-95. PubMed PMID: 16204838. eng.

2. Orton RJ, Adriaens ME, Gormand A, Sturm OE, Kolch W, Gilbert DR. Computational modelling of cancerous mutations in the EGFR/ERK signalling pathway. BMC systems biology [Internet]. 2009 2009/10//; 3:[100 p.].

3. Padala RR, Karnawat R, Viswanathan SB, Thakkar AV, Das AB. Cancerous perturbations within the ERK, PI3K/Akt, and Wnt/β-catenin signaling network constitutively activate inter-pathway positive feedback loops. Molecular bioSystems. 2017 2017/05//;13(5):830-40. PubMed PMID: 28367561. eng.
