# Supplementary material for: Estrogen receptors promote NSCLC progression by modulating the membrane receptor signaling network: a systems biology perspective
Source: J Transl Med. 2019 Sep 11;17:308. doi: 10.1186/s12967-019-2056-3 (PMC6737693; doi:10.1186/s12967-019-2056-3)
Supplement: Supplementary file 8 — Additional file 8: Table S6. Patient cohort characteristics of all patients (N = 93). [file 12967_2019_2056_MOESM8_ESM.doc]

**Additional file 8**

**Table S6** Patient cohort characteristics of all patients (n =93).

| **Characteristics** | **Frequency** | **Percent (%)** |
| --- | --- | --- |
| **Gender** |  |  |
| Male | 50 | 53.76 |
| Female | 43 | 46.24 |
| **Age (Year)** |  |  |
| ≥ 62 | 47 | 50.54 |
| < 62 | 46 | 49.46 |
| **Location** |  |  |
| Left | 38 | 40.86 |
| Right | 54 | 58.06 |
| **Tumor size (cm3)** |  |  |
| < 32 | 45 | 48.39 |
| ≤ 32 | 48 | 51.61 |
| **Lymphatic invasion** |  |  |
| Present | 14 | 15.05 |
| Absent | 79 | 84.95 |
| **TNM stage** |  |  |
| ≥ II | 62 | 66.07 |
| < II | 27 | 29.03 |
| Unknown | 4 | 4.30 |
| **Pathological grade** |  |  |
| ≤ II | 65 | 69.89 |
| > II | 28 | 30.11 |
| **Status** |  |  |
| Survival | 22 | 23.66 |
| Death | 71 | 76.34 |
